# Twin pregnancies with uterine fibroids are not at increased risk for obstetric complications: single center cohort study

**DOI:** 10.1186/s12884-020-02908-w

**Published:** 2020-04-15

**Authors:** Mi-La Kim, Kirim Hong, Sohyun Kim, Min Jin Lee, Sung Shin Shim, Yoon-Mi Hur, Joong Sik Shin

**Affiliations:** 1 Department of Obstetrics and Gynecology, CHA Gangnam Medical Center, CHA University, 566, Nonhyeon-ro, Gangnam-gu, Seoul, 135-081 Republic of Korea; 2 College of General Education, Kookmin University, Seoul, Republic of Korea

**Keywords:** Twin pregnancy, Uterine fibroid, Previous myomectomy, Preterm labor, Preterm delivery, Pregnancy outcome

## Abstract

**Background:**

Twin pregnancies with uterine fibroid(s) (UFs) may not be at increased risk for obstetric complications compared to those without UFs. However, there was no reported comparison study with obstetric outcomes and complications of twin pregnancy after myomectomy. We aimed to compare the pregnancy outcomes in twin pregnancies with or without uterine fibroid(s), and also compared in patients with previous myomectomy history in Korean women.

**Methods:**

A cohort of twin pregnancies delivered in a single institution between January 2011 and March 2019 were retrospectively analyzed. UFs group was defined by the presence of UFs during pregnancy (≥1 fibroid, measuring ≥2 cm or multiple fibroids regardless of the size). Previous myomectomy group included patients with history of abdominal or laparoscopic or hysteroscopic myomectomy of ≥1 fibroid, measuring ≥2 cm or multiple fibroids whatever the size. Patients with monochorionic monoamniotic twins, myoma less than 2 cm in size, missed abortion or intrauterine fetal death (IUFD) of one fetus before 14 weeks, history of previous conization, and uterine anomalies were excluded. Pregnancy outcomes and obstetric complications were compared.

**Result:**

A total 1388 patients were included in this study, 191 (13.8%) had UFs and 89 (6.4%) had a history of myomectomy. Maternal age was younger in non-UFs group and primiparity was more common in UFs group (*p* < 0.001, and *p* = 0.019). No significant differences were found in the gestational age at delivery (*p* = 0.657), delivery before 37 weeks (*p* = 0.662), delivery before 34 weeks (*p* = 0.340), and sum of birth weight of twin (*p* = 0.307). There were also no statistical differences in rates of obstetrical complications, such as preeclampsia, gestational diabetes mellitus, placenta previa, placenta abruption, cerclage, small for gestational age, IUFD, postpartum hemorrhage and peripartum transfusion or ICU care. These obstetrical outcomes and complications showed no statistical differences between UFs group and previous myomectomy group.

**Conclusion:**

In patients with twin pregnancies, the presence of UFs or history of previous myomectomy did not relate to negative effects on pregnancy outcomes and obstetrical complications.

## Background

Uterine fibroid(s) (UFs) are the most common benign reproductive tumors in women, and affect 20–50% of women of reproductive age [1, 2]. UFs have been shown to be associated with obstetric complications such as preterm birth, preterm premature rupture of the membranes (PPROM), intrauterine growth restriction (IUGR), and preeclampsia in singleton pregnancies [3, 4]. However, how UFs could negatively impact pregnancy course remains poorly understood. Recently, Girault et al. argued that presence of UFs or a history of myomectomy may impair the uteroplacental interface, and consequently increase the risk of spontaneous preterm birth and vascular pathologies in singleton pregnancy [4].

Epidemiological risk factors for the development of UFs include age in premenopausal years, early age at menarche, African ancestry, obesity, and infertility [5]. Interestingly, prevalence of UFs has been shown to be lower in multiparous than in nulliparous women, suggesting that parity may be protective against the development of UFs [3, 5, 6]. Given that twin pregnancy is more common among older women, and that twin birth rates have been increasing sharply due to advances in assisted reproductive technology (ART) and the trend of delayed child bearing, it is important to examine the impact of UFs in obstetric outcomes of twin pregnancies, such as preterm labor or delivery, small for gestational age (SGA), placenta abruption, and premature rupture of membranes (PROM) [7–10]. However, to our knowledge, only two studies have investigated the influence of UFs on twin pregnancies to date [11, 12]. Stout et al. examined hospital records of 2378 twin pregnancies and found that there were no significant differences between women with and without UFs in fetal weight (SGA), preterm delivery at < 34 weeks, PROM, placenta abruption, or intrauterine fetal death (IUFD) [11]. Wang et al. also reported the same results in 153 patients [12]. These results suggest that in contrast to singleton pregnancies, twin pregnancies with UFs may not be at increased risk for obstetric complications compared to those without UFs. However, there has been no reported comparison study with obstetric outcomes and complications of twin pregnancy after myomectomy.

Therefore, the objective of this study was to compare the obstetrical outcomes and complication rate in twin pregnancies with or without fibroid(s), and also patients with previous myomectomy history in Korean women.

## Methods

### Inclusion and exclusion criteria

We performed retrospective review of patients who delivered twins in CHA Gangnam Medical Center, which is a university hospital specialized for Obstetrics and Gynecology, between January, 2011 and March, 2019. Inclusion criteria for data analysis were as follows: (1) twin deliveries at ≥22 weeks of gestational age, (2) initially triplet pregnancy reduced to twin pregnancy at the time of delivery due to missed abortion or intra-uterine fetal death (IUFD) or selective abortion of one fetus before 14 weeks, (3) IUFD of one or both fetuses after 14 weeks of pregnancy. During the antenatal ultrasound exams, the presence of UFs of the patients was evaluated and grouped. The UFs group included patients with UFs during pregnancy (≥1 fibroid measuring ≥2 cm, or multiple fibroids regardless of the size). (Group A). In case of the patients with a history of abdominal or laparoscopic or hysteroscopic myomectomy of ≥1 fibroid measuring ≥2 cm or multiple fibroids regardless of the size were grouped as the previous myomectomy group (Group B), regardless of the presence of recurrent fibroid(s). And without UF patients were considered as group C.

Exclusion criteria for data analysis were as follows: patients with (1) monochorionic monoamniotic twins, (2) missed abortion or IUFD of one fetus before 14 weeks, (3) single fibroid < 2 cm or previous surgical treatment of < 2 cm fibroid according to the patients’ previous operation records, (4) associated uterine anomalies, (5) previous history of cervical conization, and (6) foreigners because ethnic/racial differences in UFs have been shown in prior studies [13]. All twin pregnancies were managed following a uniform protocol of our medical institute. During the first trimester, we confirmed the gestational age and chorionicity by transvaginal ultrasonography. If the patient first visited our clinic after late 2nd trimester and chorionicity was unclear, we confirmed the chorionicity by pathological examination of the placenta. The study protocol was approved by the Institutional Review Board of CHA Gangnam Medical Center (GCI-19-18); informed consent requirements for the study were waived given its retrospective nature. However, we obtained written consent from two patients who had experienced uterine rupture, described in this study.

### Patient characteristics and clinical definitions

The following data were extracted from the patients’ medical records: maternal age at delivery, body mass index (BMI) at delivery, parity, previous history of preterm birth, mode of conception, gestational age at delivery, birth weight of newborns, and obstetric complications such as preterm labor, PPROM, pre-eclampsia, gestational diabetes, placenta previa, operation history of cervical cerclage due to incompetent internal os of cervix (IIOC), IUFD over 2nd trimester, small for gestational age (SGA) (defined as neonatal birth weight in the < 10th percentile for gestational age) and adapted from the definition of birth weight percentiles for gestational age presented in standard tables for dichorionic and monochorionic twin pregnancies by Ananth et al. [14], placenta abruption, postpartum hemorrhage (defined as estimated blood loss over 500 ml in vaginal delivery, and estimated blood loss over 1000 ml in cesarean delivery), peripartum transfusion, and peripartum intensive care unit (ICU) admission. The sum of birth weights of twins and weight differences were calculated in patients without IUFD.

Vaginal delivery is tried when the 1st fetus is in the vertex presentation and when no other indication of cesarean delivery is met. In cases of patients with a history of myomectomy, vaginal delivery is not prohibited when the removed myoma by laparotomy or laparoscopy had invaded less than half of the myometrium thickness. In cases of hysteroscopic myomectomy, removal of type 0 or 1 submucosal myoma is allowed for future vaginal delivery. When the operational record is not available, we consider cesarean delivery. However, since a considerable part of patients in our clinic are at advanced maternal age and conceive via IVF, most of them tend to choose elective cesarean delivery. Therefore, we excluded delivery mode as a variable.

### Statistical analysis

Statistical analyses were performed using SPSS 25.00 (IBM, Armonk, NY, USA). Descriptive data were expressed as mean ± standard deviation, median, and range. Fisher’s exact test or Chi-square test was used for analysis of categorical variables. Quantitative variables were compared by means of Mann-Whitney U test or Kruskal-Wallis test for non-normally distributed measures. A *p*-value of < 0.05 was considered statistically significant.

## Results

### Baseline characteristics of the patients

During the study period, a total of 1357 twin pregnancies met the inclusion criteria. Of those, 191 women (13.8%) were classified as with UFs (Group A), 89 women (6.4%) were into previous myomectomy group (Group B), and the remainder, 1077 (79.4%), as without UFs (Group C). The baseline characteristics of the patients were compared in Table 1. Maternal age at delivery was significantly higher in patients with UFs or previous myomectomy than those without UFs (*p* < 0.00). There was a significant difference in rate of primiparity (*p* = 0.019). However, there was no significant differences in maternal BMI, medical history (prepregnancy hypertension or diabetes), previous preterm birth, mode of conception, or chorionicity. In sub-analysis, UFs group and previous myomectomy group showed similar baseline characteristics.

**Table 1:** Comparisons of baseline characteristics between twin pregnancies in three groups

Variables	Group A Twin pregnancies with UFs (*n* = 191)	Group B Twin pregnancies with previous myomectomy (*n* = 89)	Group C Twin pregnancies without UFs (*n* = 1077)	A vs B vs C *p*-value	A vs B *p*-value	A vs C *p*-value	B vs C *p*-value
Maternal age (year)	35.2 ± 3.2	35.8 ± 2.8	33.9 ± 3.1	< 0.001	0.125	< 0.001	< 0.001
	(35,24–44)	(36, 31–43)	(34, 23–45)				
BMI at delivery (kg/m^2^)	27.0 ± 2.9	27.5 ± 3.6	26.9 ± 3.1	0.250	0.228	0.741	0.097
	(26.7, 19.3–35.2)	(27.4, 20.7–41.0)	(26.6, 18–39.3)				
Medical history, number (%)							
Prepregnancy HTN	2 (1.0%)	0 (0%)	10 (0.9%)	0.645	1.0	0.700	1.0
Prepregnancy diabetes	2 (1.0%)	0 (0%)	3 (0.3%)	0.227	1.0	0.166	1.0
Primiparity	177 (92.7%)	78 (87.6%)	917 (85.1%)	0.019	0.169	0.004	0.640
Previous preterm birth	2 (1.0%)	0 (0%)	14 (1.3%)	0.542	1.0	1.0	0.617
Mode of conception				0.104	0.201	0.527	0.034
Natural or TI, COH + TI	23 (12.0%)	6 (6.7%)	163 (15.1%)				
IUI or COH + IUI	15 (7.9%)	4 (4.5%)	86 (8.0%)				
IVF or T-ET	153 (80.1%)	79 (88.8%)	828 (76.9%)				
Chorionicity				0.370	0.185	0.327	0.542
Monochorionic	20 (10.5%)	5 (5.6%)	89 (8.3%)				
Dichorionic	171 (89.5%)	84 (94.4%)	988 (91.7%)				

*UF* uterine fibroid, *BMI* body mass index, *HTN* hypertension, *TI* timed intercourse, *COH* controlled ovarian hyperstimulation, *IUI* intrauterine insemination, *IVF* in vitro fertilization, *T-ET* thawing-embryo transfer

### Pregnancy outcomes and obstetric complications

Pregnancy outcomes and obstetric complications between those with, without UFs, and previous myomectomy were compared in Table 2. None of the fetal outcomes or obstetric complications showed a significant difference between the three groups, suggesting that UFs or previous myomectomy are not associated with increased obstetric risks in twin pregnancies. In sub-analysis, UFs group and previous myomectomy group showed no statistical differences between variable obstetric complications.

**Table 2:** Comparisons of pregnancy outcomes and obstetric complications between twin pregnancies in three groups

Variables	Group A Twin pregnancies with UFs (*n* = 191)	Group B Twin pregnancies with previous myomectomy (*n* = 89)	Group C Twin pregnancies without UFs (*n* = 1077)	A vs B vs C *p*-value	A vs B *p*-value	A vs C *p*-value	B vs C *p*-value
GA at delivery (weeks)	36.0 ± 2.2	36.4 ± 1.7	36.1 ± 2.1	0.657	0.570	0.808	0.394
	(36.9, 22.6–38.9)	(36.9, 29.1–38.6)	(36.9, 22.1–39.4)				
Sum of birth weight of twin^a^	4772.1 ± 838.9	4895.3 ± 701.0	4846.8 ± 811.5	0.307	0.278	0.136	0.866
	(4810, 1172–6920)	(5020, 2530–6670)	(4970,844, 6710)				
Weight difference of twin^a^	304.8 ± 240.1	289.8 ± 228.8	284.8 ± 227.5	0.601	0.702	0.327	0.744
	(260, 0–1310)	(250, 0–1140)	(230, 0–1390)				
Delivery < 37 weeks	103 (53.9%)	53 (60.0%)	592 (55.0%)	0.662	0.378	0.813	0.438
Delivery < 34 weeks	24 (12.6%)	6 (6.7%)	115 (10.7%)	0.340	0.142	0.451	0.282
Preterm labor	78 (40.8%)	38 (42.7%)	461 (42.8%)	0.879	0.769	0.634	1.0
Admission d/t preterm labor	48 (25.1%)	21 (23.6%)	299 (27.8%)	0.558	0.781	0.482	0.459
PPROM	30 (15.7%)	14 (15.7%)	167 (15.5%)	0.996	0.996	0.914	1.0
Pre-eclampsia	22 (11.5%)	6 (6.7%)	98 (9.1%)	0.395	0.215	0.285	0.564
Gestational diabetes	25 (13.1%)	8 (9.0%)	91 (8.4%)	0.122	0.322	0.055	0.843
Placenta previa	6 (3.1%)	5 (5.6%)	34 (3.2%)	0.455	0.321	1.0	0.214
IIOC	5 (2.6%)	3 (3.4%)	42 (3.9%)	0.678	0.712	0.532	1.0
>2nd trimester IUFD	2 (1.0%)	2 (2.2%)	16 (1.5%)	0.738	0.594	0.755	0.651
SGA (< 10%)^a^	34 (18.0%)	12 (13.8%)	165 (15.6%)	0.608	0.385	0.389	0.759
Placenta abruption	2 (1.0%)	1 (1.1%)	9 (0.8%)	0.930	1.0	0.676	0.549
Postpartum hemorrhage	10 (5.2%)	7 (7.9%)	64 (5.9%)	0.686	0.391	0.867	0.485
Peripartum transfusion	16 (8.4%)	10 (11.2%)	109 (10.1%)	0.695	0.443	0.512	0.716
Peripartum ICU care	23 (12.0%)	5 (5.6%)	124 (11.5%)	0.220	0.095	0.807	0.112

^a^IUFD cases excluded (Group A, *n* = 189; Group B, *n* = 87, Group G, *n* = 1061). *GA* gestational age, *IUFD* intrauterine fetal death, *d/t* due to, *PPROM* preterm premature rupture of membranes, *IIOC* incompetent internal os of cervix, *SGA* small for gestational age, *ICU* intensive care unit

### Sub-analysis according to the size of UFs

Since our study included relatively small sized UFs in Group A (≥2 cm or multiple fibroids regardless of the size) sized UFs, we additionally analyzed the data of pateitns with larger sized UFs. According to Shavell et al. study suggested a 5 cm cut-off in singleton pregnancy [15], in Table 3, we conducted a sub-analysis according to the size of UFs (≥5 cm vs < 5 cm vs no UFs). Similarly to Table 1, maternal age and primiparity showed significant differences between the groups (*p* < 0.001 and 0.015), but there were no significant differences in pregnancy outcomes and obstetrical complications regarding the size of UFs.

**Table 3:** Comparison of baseline characteristics and pregnancy outcomes in twin pregnancies with large (≥5 cm), small (< 5 cm), or without uterine fibroid(s) (UFs)

Variables	Group A Twin pregnancies with ≥5 cm UFs (*n* = 32)	Group B Twin pregnancies with < 5 cm UFs (*n* = 159)	Group C Twin pregnancies without UFs (*n* = 1077)	A vs B vs C *p*-value	A vs B *p*-value	A vs C *p*-value	B vs C *p*-value
Maternal age (year)	34.9 ± 3.7	35.2 ± 3.1	33.9 ± 3.1	< 0.001	0.279	0.263	< 0.001
	(34, 30–44)	(35, 24–43)	(34, 23–45)				
BMI at delivery (kg/m^2^)	27.0 ± 3.2	27.0 ± 2.8	26.9 ± 3.1	0.886	0.652	0.646	0.864
	(26.7, 19.3–34.1)	(26.6, 20.6–35.2)	(26.6, 18–39.3)				
Medical history, number (%)							
Prepregnancy HTN	0 (0%)	2 (1.3%)	10 (0.9%)	0.789	1.0	1.0	0.660
Prepregnancy diabetes	0 (0%)	2 (1.3%)	3 (0.3%)	0.173	1.0	1.0	0.127
Primiparity	31 (96.9%)	146 (91.8%)	917 (85.1%)	0.015	0.472	0.073	0.027
Previous preterm birth	0 (0%)	2 (1.3%)	14 (1.3%)	0.810	1.0	1.0	1.0
Mode of conception				0.609	0.470	0.601	0.438
Natural or TI, COH + TI	5 (15.6%)	18 (11.3%)	163 (15.1%)				
IUI or COH + IUI	1 (3.1%)	14 (8.8%)	86 (8.0%)				
IVF or T-ET	26 (81.3%)	127 (79.9%)	828 (76.9%)				
Chorionicity				0.113	0.113	0.049	0.760
Monochorionic	6 (18.7%)	14 (8.8%)	89 (8.3%)				
Dichorionic	26 (81.3%)	145 (91.2%)	988 (91.7%)				
GA at delivery (weeks)	36.2 ± 1.8	36.0 ± 2.3	36.1 ± 2.1	0.867	0.833	0.889	0.602
	(36.9, 31.3–38.3)	(36.9, 22.6–38.9)	(36.9, 22.1–39.4)				
Sum of birth weight of twin^a^	4794.1 ± 628.9	4767.6 ± 877.2	4846.8 ± 811.5	0.259	0.837	0.361	0.156
	(4810, 3410–5950)	(4810, 1172–6920)	(4970,844, 6710)				
Weight difference of twin^a^	331.6 ± 236.3	299.3 ± 241.2	284.8 ± 227.5	0.401	0.383	0.202	0.587
	(255, 0–990)	(260, 10–1310)	(230, 0–1390)				
Delivery < 37 weeks	19 (59.4%)	84 (52.8%)	592 (55.0%)	0.767	0.563	0.719	0.670
Delivery < 34 weeks	4 (12.5%)	20 (12.6%)	115 (10.7%)	0.744	1.0	0.769	0.495
Preterm labor	15 (46.9%)	63 (39.6%)	461 (42.8%)	0.660	0.555	0.718	0.492
Admission d/t preterm labor	9 (28.1%)	39 (24.5%)	299 (27.8%)	0.691	0.660	1.0	0.446
PPROM	5 (15.6%)	25 (15.7%)	167 (15.5%)	0.997	1.0	1.0	0.907
Pre-eclampsia	3 (9.4%)	19 (11.9%)	98 (9.1%)	0.518	1.0	1.0	0.247
Gestational diabetes	4 (12.5%)	21 (13.2%)	91 (8.4%)	0.121	1.0	0.346	0.055
Placenta previa	0 (0%)	6 (3.8%)	34 (3.2%)	0.537	0.592	0.621	0.632
IIOC	1 (3.1%)	4 (2.5%)	42 (3.9%)	0.679	1.0	1.0	0.504
>2nd trimester IUFD	0 (0%)	2 (1.3%)	16 (1.5%)	0.742	1.0	1.0	1.0
SGA (< 10%)a	5 (15.6%)	29 (18.2%)	165 (15.6%)	0.646	0.806	1.0	0.351
Placenta abruption	0 (0%)	2 (1.3%)	9 (0.8%)	0.750	1.0	1.0	0.642
Postpartum hemorrhage	3 (9.4%)	7 (4.4%)	64 (5.9%)	0.510	0.376	0.436	0.583
Peripartum transfusion	4 (12.5%)	12 (7.5%)	109 (10.1%)	0.525	0.315	0.559	0.390
Peripartum ICU care	2 (6.3%)	21 (13.2%)	124 (11.5%)	0.521	0.378	0.570	0.511

^a^IUFD cases excluded (Group A, n = 32; Group B, *n* = 157, Group G, n = 1061). *BMI* body mass index, *HTN* hypertension, *TI* timed intercourse, *COH* controlled ovarian hyperstimulation, *IUI* intrauterine insemination, *IVF* in vitro fertilization, *T-ET* thawing-embryo transfer, *GA* gestational age, *IUFD* intrauterine fetal death, *d/t* due to, *PPROM* preterm premature rupture of membranes, *IIOC* incompetent internal os of cervix, *SGA* small for gestational age, *ICU* intensive care unit

### Experiences of uterine rupture in twin pregnancies

During the study period, we experienced two cases of uterine rupture in twin pregnancies. One patient was with a history of laparoscopic myomectomy of 3.5 cm sized deep intramural myoma with endometrial compression and 2.8 cm sized intramural myoma on the posterior corpus. Seven months later, she had conceived twin via thawing-embryo transfer. At 30 + 4 weeks of gestation, she experienced sharp abdominal pain and was diagnosed with preterm labor. She was admitted and tocolytics were administered. At 31 + 4 weeks of gestation, with a sudden deceleration of fetal heart rate on the cardiotocography, the patient underwent emergency cesarean section and about 5 cm sized rupture site was found on the posterior corpus of the uterus. The other patient was with a history of laparoscopic right cornual resection due to right cornual pregnancy. She had conceived via in vitro fertilization (IVF), 2 years after the surgery, and at 31 + 6 weeks of gestation, visited the emergency room due to low abdominal pain. She was diagnosed with preterm labor and tocolytics were administered. However, at 33 + 5 weeks of gestation, the patient complained a sudden severe abdominal pain, and cardiotocography showed fetal deceleration of one fetus, which led to emergency cesarean section and ruptured right cornus of the uterus was confirmed.

## Discussion

Our study indicates that twin pregnancies with UFs, even with those of larger sized as more than 5 cm, do not significantly increase the risk for obstetric complications or adverse pregnancy outcomes as compared to those without UFs. Specifically, we did not find any significant differences between twin pregnancies with, without UFs, and previous myomectomy in gestational age at delivery, sum of birth weight of twins, preterm delivery and labor, PPROM, pre-eclampsia, gestational diabetes, placenta previa, IIOC, >2nd trimester IUFD, SGA, placenta abruption, postpartum hemorrhage, peripartum transfusion, or peripartum ICU admission.

In singleton pregnancies the most common neonatal morbidity associated with UFs is known to be preterm delivery [16, 17]. However, the rates of preterm delivery (delivery < 37 weeks and < 34 weeks) between twin pregnancies in our three groups of study patients showed no differences, providing a strong evidence that in contrast to singleton pregnancies, UFs is not associated with complications in twin pregnancies. Our results are consistent with the findings from the study by Stout et al. [11]. As Stout et al. examined twin pregnancies in a predominantly white Caucasian, our results suggest that negligible associations between UFs and outcomes of twin pregnancies may be generalized to East Asian women. However, UFs develop earlier, are larger, and more symptomatic in African than in European American women [13]. Thus, our findings may not be generalized to women with African ancestry. Given that twin birth rates are high in Africans, it would be important for future studies to explore the impact of UFs in twin pregnancies in women with African ancestry [18].

The prevalence of UFs found in our study was higher than the rates found in study by Stout et al. [11]. Note that our patients were much older than other samples, and that primiparity was predominant (85%) in our sample, which may be responsible for the high incidence of UFs in our sample, which is consistent with previous studies showing that UFs tends to increase with age [5].

We also confirmed that a history of myomectomy does not affect the complication rates in twin pregnancies. To investigate the impact of myomectomy, we compared those who underwent myomectomy (*n* = 89), those who did not (*n* = 191), and those who had no UFs (*n* = 1077) in baseline characteristics and pregnancy outcomes. As indicated in Table 2, none of the differences between operated and unoperated groups attained statistical significance.

Moreover, in our study patients, only 14% of pregnancies were conceived naturally or via timed intercourse with or without ovarian hyperstimulation. Most of the patients were conceived by ART with any reasons. The possibility of UFs and subfertility has been considered in gynecologic field [19]. Pritts et al. suggested that the presence of UFs at any location showed decreased clinical pregnancy, implantation, ongoing pregnancy/live birth rate and increased spontaneous abortion rate in their systematic review [19]. In their sub-analysis by location, subserosal fibroid(s) had no differences on fertility outcomes, and myomectomy did not change these outcomes; intramural fibroid(s) appeared to have decreased fertility and increased pregnancy loss, and myomectomy did not significantly increase the clinical pregnancy and live birth rates; however, submucosal component led to decreased clinical poregnancy and implantation rates, and removal of submucosal fibroid(s) appeared to improve fertility [19]. Especially, in infertile women, submucosal myoma and deep intramural myoma with distorted endometrial cavity are considered to benefit from myomectomy [19, 20]. As a result, the incidence of myomectomy could be increased in older age group due to fibroid associated menorrhagia, pain, compression symptoms and subfertility. Girault et al. suggested that the risk of preterm birth was persisting after myomectomy in singleton pregnancy due to irreversible damage to myometrium, and potential dysregulation of hormone and inflammatory cytokines [4]. However, opposite results are demonstrated in our study: the presence of UFs or previous myomectomy does not add on to the adverse effects on pregnancy outcomes and obstetric complications in case of twin pregnancies.

However, considering the two cases of uterine rupture in our study, unnecessary myomectomy should be avoided. In one retrospective review of 19 cases of uterine rupture after laparoscopic myomectomy, the authors recommended multilayered closure of the myometrium and limited use of electrocautery for prevention of uterine rupture [21]. Moreover, during antenatal care of women with scarred uterus, symptoms such as sharp low abdominal pain should be urgently managed with alert, considering the possible occurrence of uterine rupture.

It is well known that twin pregnancies carry increased risks for obstetric complications. Especially, early uterine distension is thought to induce preterm labor in women with twin pregnancies [22]. In explaining no significant association between obstetric outcomes and UFs in twin pregnancies, Stout et al. proposed that more frequent check-up, early uterine distension, and planned early delivery in twin pregnancies might have mitigated adverse effects that could be attributable to fibroid tumors detected in singleton pregnancies [11].

A major strength of our study is the inclusion of a large cohort of patients. And this study is the first comparison study with obstetric outcomes and complications of twin pregnancy after myomectomy. However, there are several limitations in our study. First, the diverse characteristics of UFs including location, and numbers were not compared. In Table 3, we proposed that the size of UFs (large with ≥5 cm vs small with < 5 cm, and no UFs) in twin pregnancies is not associated with adverse obstetric outcomes. However, due to the relatively small sample size, we were not able to perform analysis by other characteristics of UFs. In two previous retrospective studies, preterm delivery was more common in multiple fibroids [23, 24]. And in one study, fibroids in the lower part of uterus showed higher cesarean section rate, postpartum hemorrhage, greater estimated blood loss, and higher rates of admission for fibroid related pain [23]. Secondly, we did not evaluate the rate of the first trimester pregnancy loss. Many clinicians and patients have been interested in the UFs affecting the implantation failure and early pregnancy loss in first trimester. According to a systemic review by Klatsky et al., in singleton pregnancies, the presence of UFs itself raised the rate of early pregnancy loss by 2.9 times, and Pritts et al., also reported that it was raised by 1.7 times regardless of the location of UFs [17, 19]. However, we could not evaluate the impact of UFs in the first trimester of twin pregnancies because many of our patients were transferred to our hospital after confirmation of twin pregnancies. Finally, generalizability of the results may be limited because the data were drawn from a single maternity hospital in Seoul, South Korea, and power analysis was not used. Future prospective studies are required to overcome these limitations of the important subject.

## Conclusion

In conclusion, our study confirmed that in twin pregnancies, the presence of UFs or previous myomectomy is not related to adverse outcomes of pregnancy or obstetric complications. Considering the recent advanced ART and subsequent increase of twin pregnancies, our data could be encouraging to the patients who suffer from infertility with UFs or previous myomectomy and are afraid of conceiving twin pregnancies after ART procedure. For confirmation of these results, additional large-scaled multicenter studies may be required.

## Data Availability

Data will be available upon reasonable request from the corresponding author. However, the data cannot be made public to maintain women’s privacy and legal reasons as it contains private health information along with identifiers.

## Abbreviations

UFs: Uterine fibroid(s)
PPROM: Preterm premature rupture of membranes
IUGR: Intrauterine growth restriction
ART: Assisted reproductive technology
PROM: Premature rupture of membranes
IUFD: Intrauterine fetal death
BMI: Body mass index
IIOC: Incompetent internal os of cervix
SGA: Small for gestational age
ICU: Intensive care unit
